# Understanding socio-economic determinants of childhood mortality: a retrospective analysis in Uganda

**DOI:** 10.1186/1756-0500-4-484

**Published:** 2011-11-09

**Authors:** Fred Nuwaha, Juliet Babirye, Olico Okui, Natal Ayiga

**Affiliations:** 1 Makerere University School of Public Health, P.O. Box 7072, Kampala, Uganda; 2 Institute of Statistics and Applied Economics, Makerere University, P.O. Box 7072, Kampala, Uganda

## Abstract

**Background:**

Teso sub-region of Eastern Uganda had superior indices of childhood survival during the period 1959 to 1969 compared to the national average. We analysed the reasons that could explain this situation with a view of suggesting strategies for reducing childhood mortality.

**Methods:**

We compared the childhood mortalities and their average annual reduction rate (AARR) of Teso sub-region with those of Uganda for the period 1959 to 1969. We also compared indicators of social economic well being (such as livestock per capita and per capita intake of protein/energy). In addition data was compared on other important determinants of child survival such as level of education and rate of urbanisation.

**Findings:**

In 1969 the infant mortality rate (IMR) for Teso was 94 per 1000 live births compared to the 120 for Uganda. Between 1959 and 1969 the AARR for IMR for Teso was 4.57% compared to 3% for Uganda. It was interesting that the AARR for Teso was higher than that that of 4.4.% required to achieve millennium development goal number four (MDG4). The rate of urbanisation and the level of education were higher in Uganda compared to Teso during the same period. Teso had a per capita ownership of cattle of 1.12 compared to Uganda's 0.44. Teso sub region had about 3 times the amount of protein and about 2 times the amount of calories compared to Uganda.

**Conclusions:**

We surmise that higher ownership of cattle and growing of high protein and energy foods might have been responsible for better childhood survival in Teso compared to Uganda.

## Background

The socio-economic condition of the population is a major determinant of childhood survival at individual, household and community level [1,2]. Social economic conditions influence the risk of childhood mortality by influencing intermediate or proximate variables such maternal factors, environmental contamination, nutrient deficiency, injury and personal illness control [3-8]. Childhood mortality is thus multi-factorial in causality, may have long latency periods between exposure and manifestation and thus the need for a multidisciplinary approach to understanding the causes and methods of alleviating childhood mortality is clear [9,10]. Such an approach is also useful to understand the mortality disparities say between geographical regions and between different segments of the population [10]. Therefore an in depth investigation to connect ecological or socio-economic factors that may influence specific proximate determinants can give policy makers insights into development strategies that could reduce the differentials in childhood mortality [9-11]. Such an understanding could also lead to implementation of strategies/programmes aimed at a rapid decline in childhood mortality especially in low income countries where the rates are very high [11]. This approach may thus enable an acceleration of reduction in childhood mortality necessary to achieve millennium development goal number four (MDG4) especially in areas that face an uphill task in achieving the goal such as Uganda [12].

In this report we compared the childhood mortality in Teso sub-region of Uganda to the Ugandan national averages. We also analyse why Teso sub-region of Uganda appeared to have superior childhood survival indices compared to Uganda during the period 1959-1969. The period 1959 to 1969 was chosen because it was the most stable politically and most prosperous time during the history of Uganda [13]. Besides data for comparison during the said time was available (e.g.) from the 1959/69 census as well as from the 1962/63 and 1967/68 agricultural censuses [14-16].

## Methods

### Setting

During the period 1959-1969, Uganda was classified as a low income country whose gross domestic product (GDP) per capita was about 656 United States Dollars [17]. The total area of Uganda is approximately 241038 km^2^. However, the land surface covers only 197100 km^2^, as open water resources such as the Lake Victoria take about 18% of the country. The population of Uganda in 1969 was about 9.5 million people who were predominantly rural dwellers (about 94%) and rapidly growing at an annual rate of 3.9% [15]. More than 95% of the total population were engaged in subsistence agriculture which comprised a large variety of both crops and livestock products. Agriculture formed the backbone of the economy and contributed over 80% to the GDP and over 90% to the export revenue. The main cash crops were coffee, tea, cotton and tobacco whereas the main food crops were bananas, maize, millet, cassava, beans, groundnuts and simsim.

Teso sub-region is found in the eastern part of Uganda covering a land area of 11060 km^2 ^and had about 6% of Uganda's population. Since 1910, Teso has had a superior economy, dietary and general wellbeing compared to the Uganda national averages [14,15,18]. Indeed in the 1960s the wellbeing of Teso was comparable to that of Kampala city and its surrounding peri-urban prosperous county of Kyadondo (now Wakiso district) [15]. In 1959 and 1969 Uganda was divided into 17 administrative districts. In terms of infant mortality rate (IMR) Teso was ranked third of the 17 districts. In 1959 the IMR for Teso was 150 per 1000 live births compared to 160 per 1000 live births for Uganda. In 1969 the IMR for Teso was 94 compared to 120 for Uganda.

The major predictors of improved childhood survival in Uganda at population level in Uganda during the period 1959-1969 were living in an urban environment as well high level of maternal education [14,15]. Current data also indicates that urban environment, high maternal education, wealth index as well as low total fertility (reflected in birth spacing) are important for child survival [19].

### Design

We compared the childhood mortalities and average annual reduction rates (AARR) of Teso sub-region with those of Uganda for the period 1959 to 1969. The childhood mortalities compared were infant mortality rate (IMR), child mortality rate (CMR) and under-five mortality rate (U5MR). The IMR is the probability of dying between birth and the first birth day. The CMR is the probability of dying between exact one age and the fifth birth day whereas the U5MR is the probability of dying between birth and fifth birth day. The U5MR and the IMR are expressed as deaths per 1000 live births and the CMR is expressed as deaths per 1000 children surviving to the first birth day. Because data on CMR was not available for the 1959 censuses we used the IMR for the calculation of AARR.

We also compared proxy indicators of social economic well being (such as livestock per capita area under major crops) for Teso sub region and for Uganda. In addition data was compared on other important determinants of child survival such as level of education, rate of urbanisation and the total fertility rate.

### Data sources

The sources of data for childhood mortality in this study were derived from the 1959 and 1969 Uganda national censuses [14,15]. Data on urbanization, level of education and on total fertility is also available in 1959 and 1969 censuses reports for the country. Objective and reliable data on agriculture (crops and livestock) was obtained from the 1967/68, agricultural sample survey [16]. This survey built on the 1963/64 censuses on Agriculture. Although routine agricultural reporting services started in Uganda during the colonial times (in 1950s) where district officers collected and provided data on crops and live stock, these data were commonly incomplete and inaccurate and could therefore not be relied on for this analysis.

### Ethical considerations

The Uganda National Council for Science and Technology (UNCST) and the Makerere University Institute of Public Health (MUIPH) institutional review board independently approved the study. Prior to data collection permission was sought from the relevant Uganda government authorities.

## Results

The trends of childhood mortalities in Teso sub-region compared to the Uganda national average are shown in Table 1. Teso had lower childhood mortalities compared to Uganda. This difference was found between both child mortality and infant mortality rate but was more marked for child mortality (see percentage differences in parentheses of Table 1.). Between 1959 and 1969, the average annual reduction rate (AARR) of Infant mortality rate for Teso of 4.57% was superior to that of Uganda of 3%.

**Table 1:** Childhood mortalities in Teso sub-region compared to Uganda for the period 1959-1969

Indicator	Teso	Uganda	Difference
1969 Census			

U5MR	158	215	+57 (36%)

CMR	66	90	+24 (36%)

IMR	94	118	+24 (26%)

1959 Census			

IMR	150	160	+10 (6.3%)

references [14,15]

In Table 2 we compared maternal education, urban environment as well as total fertility for Teso and Uganda during 1959-1969. As can be seen, the rates of urbanisation and of maternal education were lower in Teso compared to Uganda. The total fertility for Teso was much lower than that of Uganda.

**Table 2:** Total fertility, urbanization and education status of Teso and Uganda 1959-1969

Indicator	1959		1969	
	
	Teso	Uganda	Teso	Uganda
Total fertility	3.2	5.1	5.1	7.2

Population density (square km)	41	33	52	48

Urbanisation (%)	2.5	3.8	2.1	6.7

Education (ever been to school among those aged 16-45 years)				

Male (%)	41	43	45	47

Female (%)	14	17	17	21

references [14,15]

Because Uganda was and still is predominantly an agricultural country we compared agricultural production in Teso to that in Uganda as a proxy for wealth index. The results of these comparisons are shown in Tables 3, 4, and 5. Table 3 shows that about 15% of the national herd of cattle in 1968 were in Teso. Other indicators such as percent of households with cattle, per capita ownership of cattle and use of cattle for traction are much superior in Teso compared to Uganda.

**Table 3:** Cattle ownership and use in traction in Teso sub region compared to Uganda in 1967/68

Indicator	Teso	Uganda
Population (people)	570628	9548847

Number of cattle	627690	4201492

Cattle per capita	1.12	0.44

Percent of households with cattle	95	35

Percent of households using animal traction	93	32

reference [16]

**Table 4:** Area under major crops in Teso (area 11060 km^2^) compared to Uganda (area 197100 km^2^) in 1967/68

Crop	Teso	Uganda
	
	Km^2 ^(%)	Km^2 ^(%)
Finger millet	1873(17)	5109(2.6)

Sorghum	462(3.2)	23309(1.3)

Ground nuts	515(4.2)	1698(0.9)

Cowpeas	1077(9.7)	1766(1)

Bananas	57(0.5)	14711(7.5)

Cassava	135(0.9)	2243(1.3)

Maize	290(2.6)	3002(1.5)

Beans	65(0.6)	2195(1.1)

Simsim	104(0.9)	780 (0.4)

Sweet Potato	168 (1.5)	1143 (0.6)

reference [16]

**Table 5:** Composition of major foods in Uganda and per capita of protein and calories derived from the major foods in Teso compared to Uganda in 1968

Food composition of major foods in Uganda (per 100 g of edible portion)				
**Food**	**Calories**	**Protein (g)**	**Iron (Mg)**	

Finger millet	346	8.7	4.0	

Sorghum	354	10.2	4.1	

Ground nuts	577	27.1	2.5	

Cowpeas	330	22.4	5.0	

Bananas	100	1.5	0.4	

Cassava	350	1.8	2.0	

Maize	354	9.0	2.5	

Beans	330	19.5	8.0	

Simsim	593	20.1	9.8	

Sweet Potato	166	1.3	1.2	

Per capita of protein and calories derived from the major foods				

Food	Protein		Calories	
	
	Teso	Uganda	Teso	Uganda

Finger millet	53.40	8.46	2292	497

Sorghum	22.0	5.13	704	164

Ground nuts	27.20	6.03	571	126

Cowpeas	38.00	3.51	570	53

Bananas	0.30	3.24	26	324

Cassava	3.70	3.96	814	854

Maize	7.10	7.21	258	261

Beans	3.90	12.25	55	172

Simsim	3.30	1.53	99	46

Sweet Potato	4.80	2.70	365	205

Others	0	1.83	708	123

Total	163.70	55.85	6462	2825

reference [20]

The area under major food crops is shown in Table 4. In Teso 4746 km^2 ^(33%) of the land area was under food crops compared to 34902 (19.5%) for Uganda. The major crops grown in Teso were finger millet, sorghum, ground nuts and cow peas whereas the major crops grown in Uganda were bananas, cassava, sweat potatoes, maize and beans.

Table 5 shows the energy and protein content of major food crops that were grown in Uganda and how their availability varied for Teso compared to Uganda. Because the crops grown in Teso sub region were of high protein and high energy density the sub region had about 3 times the amount of protein and about 2.3 times the amount of calories compared to Uganda. These figures did not take into effect animal's sources of protein and energy but given the fact that Teso sub region had about 2.5 times the number of cattle compared to Uganda, one may assume that the diet in Teso was even richer than the average diet in Uganda.

## Discussion

These data show that childhood survival in Teso sub-region for the period 1959-1969 were superior compared to Uganda. The AARR between 1959 and 1969 of IMR (4.57%) for Teso was much higher than for Uganda (3%). It was also interesting to note that the AARR for Teso was more than the 4.4% required to attain MDG4 [12].

Our analyses also show that level of urbanisation was higher in Uganda compared to Teso sub-region. The level of education was also slightly better in Uganda compared to Teso. On the other hand total fertility was lower in Teso compared to Uganda. Furthermore, ownership of cattle and acreage under crops was much higher in Teso. Moreover, the crops grown in Teso were more nutritious being richer in protein, energy and in iron.

Because urbanisation and education were more favourable in Uganda compared to Teso we surmised that these indicators could not be responsible for the superior child survival in Teso. We instead hypothesized that the favourable indices related to ownership of cattle, differences in acreage under crops and in total fertility observed in Teso could be the ones responsible for the superior child survival. The low total fertility in Teso compared to Uganda has been linked to a cultural norm of prolonged breastfeeding that was estimated to last more than 2 years [15].

In this study we used ownership of cattle, land area under crops and availability of per capita protein and energy foods as proxy measures of socioeconomic wellbeing. This is because conventional measures of social economic status such GDP per capita and the gini coefficient were not available for Teso sub-region and for Uganda. However, such indicators are major determinants of social-economic status especially in agricultural communities of Uganda [21,22].

Besides this analyses were based on retrospective data collected in censuses. It was therefore not possible to make comparisons regarding other causes of childhood survival such as access to health services.

The superiority of socio-economic characteristics of Teso sub-region were based on a peculiar farming system whose advantages could not be replicated anywhere in Uganda [18,20]. The most distinguishing characteristic of the Teso farming system was the keeping of livestock and growing of high energy-protein crops, the two components being intricately integrated. This farming system was based on the use of ox-drawn implements, in a finger millet-cotton economy under a fairly high population and livestock density. Due to the use of animal draught, the system had the highest cultivated land per capita in the whole country, estimated at 8 acres per capita.

Thus the superior childhood survival indices in Teso sub region could be explained from several perspectives. First are the advantages of a cattle based mixed type of farming. Data from Uganda that analysed determinants of childhood mortality at household level has demonstrated ownership of cattle as major determinant of child survival [22]. The Livestock in Teso sub-region provided multiple benefits such as manure for improving soil fertility, animal traction and as source of cash all of which improves community wellbeing. Besides, because of livestock animal based traction men could take part in crop production through opening land. This would allow women particularly the pregnant and lactating women time for rest and more time to care for the children. Contemporary research point to the fact that as women are engaged in increased production relative to rearing of children, the benefits of increased production do not directly translate into well being of children [23]. Moreover, the use of animal draught, lead to higher productivity and easier opening of virgin land for agriculture meaning that fallowing was possible thereby allowing previously used land to rest and regain fertility [18].

Second the crops grown in Teso sub region had a very much higher density of protein and energy than those of Uganda meaning that malnutrition in Teso sub-region was much lower than the national average [18,20]. The food in Teso sub region was also more likely to be complete with essential amino-acids such as lysine and methionine [20,24] Furthermore, the major food crops produced in Teso (especially millet and groundnuts) were more likely to be stored for longer periods of time without being destroyed compared to major foods for the rest of Uganda. This increased food security in Teso and reduced the risk of food shortages [18,20,24].

## Conclusion

The data suggest that mixed farming based on ownership of cattle, growing of high protein/high energy foods and semi-mechanization (as use of animal traction) may be an excellent combination to improve wellbeing of rural populations and accelerate the decline in childhood mortalities.

## Competing interests

The authors declare that they have no competing interests.

## Authors' contributions

FN contributed to the study concept, and design, analysis of the data, writing and editing of the paper. JB contributed to collection of data, analysis and in writing and editing of the paper. OO contributed in analysis and in writing of the paper. NA contributed to data analysis and writing of the paper.
